# How Do Avalanche Dogs (and Their Handlers) Cope with Physical Exercise? Heart Rate Changes during Endurance in a Snowy Environment

**DOI:** 10.3390/ani12020168

**Published:** 2022-01-11

**Authors:** Laura Menchetti, Martina Iaboni, Michele Matteo Santoro, Gabriella Guelfi, Silvana Diverio

**Affiliations:** 1 Department of Agricultural and Food Sciences, Alma Mater Studiorum—University of Bologna, 40127 Bologna, Italy; laura.menchetti7@gmail.com; 2 Veterinary Consultant, Via Colle del Bagno 52/a, 03029 Veroli, Italy; martinaiabby@gmail.com; 3 Italian Military Corp of Guardia di Finanza, Via Lungolago 46, 06061 Castiglione del Lago, Italy; Santoro.MicheleMatteo@gdf.it; 4 LEBA (Laboratory of Ethology and Animal Welfare), Department of Veterinary Medicine, University of Perugia, Via San Costanzo 4, 00126 Perugia, Italy; gabriella.guelfi@unipg.it

**Keywords:** working dogs, search and rescue dogs, physical efforts, heart rate monitors, dog welfare, endurance, animal-based measures, altitude, gradient

## Abstract

**Simple Summary:**

Search and rescue (SAR) dogs are irreplaceable support in natural disasters. Not only are these animals required to have optimal scenting capabilities, but they are also required to have excellent physical conditions. Implementing protocols to monitor their fitness would help to optimize their performance and welfare. This study evaluated heart rate (HR) changes in avalanche SAR dogs and handlers during a 5.5 km endurance exercise in the snow, at an altitude of approximately 2000 m.a.s.l., reflecting their usual working and workload conditions. Dogs’ and handlers’ HR and activities were monitored by a global positioning satellite (GPS)/HR system. Factors influencing dogs’ and handlers’ HR changes and their possible correlation were investigated. As expected, the dog’s HR changes during the endurance activity were affected by speed, gradient, altitude, and time. The handlers’ HR changes differed and were not correlated with those recorded in the dogs. Thus, SAR handlers may not perceive the physical stress of their dog in real-time. Findings indicate that GPS/HR monitoring systems could be utilized in field conditions for monitoring SAR units’ physical fitness. A “fitness index” could be developed by incorporating HR and velocity measurements in order to target training strategies and indicate risk factors for physical distress in working dogs.

**Abstract:**

This study aimed to assess the heart rate (HR) responses of avalanche SAR dogs and handlers under working field conditions. Thirteen SAR units (dogs and handlers) performed an exercise (*Endurance*) consisting of approximately 5.5 km of rough tracks through deep snow, at an altitude of 1991–2250 m.a.s.l. The exercise was repeated twice for each of the two different tracks. Both handlers and dogs were equipped with a global positioning satellite/heart rate (GPS/HR) system (Polar^®^). Multivariable models were used to evaluate the effects of environmental (i.e., gradient, altitude, track, and time) and intrinsic (i.e., speed, repetition, and breed) factors on changes from baseline HR (Δ%HR). The dog’s Δ%HR was greater in the flat and uphill compared with downhill, and increased progressively as the speed increased (*p* < 0.001). Moreover, it rose at altitudes above 2100 m.a.s.l. and peaked after 30 min of the *Endurance* activity (*p* < 0.01). These findings indicated that HR monitors could be a valuable tool to contribute to the evaluation of avalanche dogs’ fitness in their real working environment. In contrast, the lack of correlation between the dogs’ and handlers’ HR changes suggests that handlers might not perceive the physical conditions of their dog in real-time. Thus, implementing protocols to monitor avalanche SAR dogs’ fitness using a GPS/HR monitoring system could help handlers to tailor the training and workload and to detect the risk factors for physical distress of working dogs.

## 1. Introduction

Search and rescue (SAR) dogs are still irreplaceable support in natural disasters [1,2,3]. They can cover a large area very fast, such as a 1 ha avalanche in 30 min, whereas a probe line of 20 people takes approximately 4 h [2]. Avalanche SAR dogs must have optimal scenting capabilities and be in excellent physical condition to deal with activities related to their work environment. Sometimes, professional rescue units need to reach the avalanche or earthquakes site in a limited time by walking or ski mountaineering on fresh snow. Indeed, a limited transport capacity is a major problem for rescue teams [2]. In these settings, both the handler and dog are subjected to physical and psychological stress that might compromise their search performance [1]. Fatigue can severely compromise SAR dogs’ concentration and their olfactory ability, in addition to their welfare [3,4,5]. Therefore, identifying parameters that may be markers of fitness or indicators of the ability to cope with psychophysical strain in SAR dogs could be a tool to improve their training and performance, as well as their welfare.

The level of training, health status, and physical fitness are often assessed using heart rate (HR) responses to speed, and Polar^®^ human heart rate monitors are tools that are used also in non-human animals [6,7,8]. In dogs, this device has mainly been employed to monitor HR changes during treadmill exercise tests [9,10,11]. The Polar^®^ system is advantageous over electrocardiography (ECG) as it is more animal friendly and substantially more economical [6]. It is portable and allows for the monitoring of moving animals, not only in treadmill testing, but also in field conditions. It can also have an integrated GPS unit that provides information on the speed and position. The Polar^®^ system, therefore, seems to be an ideal tool to quantify the actual workload of animals used for work, sport, and recreation, as treadmill testing does not reproduce the physiological changes nor the biomechanics of locomotion on natural environments [12]. However, the applications of the Polar^®^ in the field trials are not yet widespread, and there are few reports on working dogs. Telemetric heart rate monitoring systems have previously been used to monitor the HR of working cattle dogs during actual mustering exercises [13] and, more recently, of sled dogs undergoing training for mid-distance races [14]. However, to our knowledge, no studies involved avalanche dogs.

The physiology of avalanche dogs generally receives little attention, and this is surprising considering their significant social impact. Two studies investigated the stress-related responses and behavioral strategies during simulated avalanche searches [3,15]. However, there are no reports on modifications associated with endurance exercise, which is an integral part of the daily activities of avalanche dogs. As a result, there is a gap in animal-based measures (ABMs) that could be used to adjust their training and workload, as well as to assess their welfare. On the other hand, the difficulty of monitoring animal physiological parameters (e.g., HR, body temperature, blood lactate or creatine kinase level) in the environmental conditions in which avalanche dogs work must be emphasized. Climatic and logistic conditions at high altitudes make the use of specific and expensive devices, such as the Holter monitor, as well as the collection of blood samples, tricky. Physiological parameters, moreover, could be influenced by many factors that are difficult to standardize. In fact, unlike tests in controlled environments, in the high mountains, the environmental temperature and ventilation, as well as the soil conditions, are changeable and unstable. Finally, in a real work environment, the handler could influence the dog’s physiological condition in several ways. A role, for example, could be played by the approach of the handler during and following a trial, including affiliative or punitive behaviors [16,17] and his personality or moods [18,19]. Some authors have also hypothesized a sort of synchronization of emotional states capable of influencing the dog’s physical and cognitive performance [17,20]. The handler, moreover, can monitor the physical responses of his dog closely and in real-time and shares activities and the work context. They could then identify the signs of distress and manage the training and workload accordingly [1]. However, to our knowledge, there are no studies on the possible associations between dogs’ and humans’ HR during endurance exercises.

This study hypothesized that the Polar^®^ system could monitor the HR in avalanche dogs in the field conditions. Thus, the Polar^®^ should capture the HR changes due to several factors, such as speed, gradient, and altitude. An association between the SAR dogs’ and handlers’ HR was also hypothesized. Monitoring the HR during an endurance exercise may be important for implementing training protocols and managing work and rest cycles. It could also represent a feasible ABM for the welfare and health assessment of avalanche dogs.

Thus, this study aimed to monitor the HR and activities (distance, speed, and altitude) of a group of avalanche SAR dogs and their handlers during endurance exercise in fresh snow by using Polar^®^ GPS/HR monitoring systems. The main effect of the characteristics of the track, gradient, speed, altitude, and breed on HR changes in both dogs and handlers were evaluated. Moreover, associations between the relative HR changes from the baseline of dogs and handlers were also quantified.

## 2. Materials and Methods

All experimental procedures in this study were authorized by the Ethical Committee of Perugia University (document number 2018-21). There is a standing agreement between the Italian Military Force of Guardia di Finanza (GdF) and the Department of Veterinary Medicine of Perugia University to allow ethical studies on GdF working dogs. This study is part of a broader collaborative project among the Italian Military Force of GdF, the Department of Veterinary Medicine of Perugia University, and the Agenzia Regionale per la Prevenzione e Protezione Ambientale del Veneto (ARPAV). The project aims to identify factors affecting the success of avalanche search and rescue missions to increase the recovery rate of buried victims.

### 2.1. Experimental Conditions

**Figure 1:**
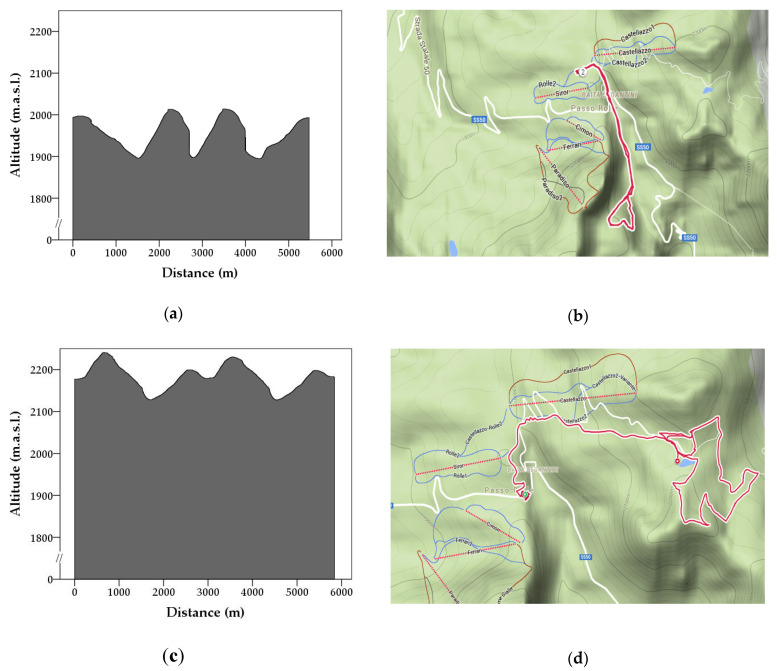
*Endurance* altitude and map routing (as produced by the Polar^®^ GPS) of Track A (panels (**a**,**b**)) and Track B (panels (**c**,**d**)).

The study was carried out in March and April 2019 during two weeks of conventional training sessions of the GdF Alpine School (SAGF) at the Passo Rolle’s snow area (Trento, Italy; 2001 m.a.s.l.). The environment air temperatures ranged from −6 to 5 °C during the first week, and from −2 to 9 °C during the second one. The humidity ranged, during the whole experimental period, between 50 and 60%, and the wind from 2 km/h to 24 km/h. Visibility was good and the sky was mostly cloudy. In this area, two *Endurance* routes (Track A and B for the first and second session, respectively) in fresh snow, approximately 5.5 km in length, were designed. Flags were used to indicate the track. Track A had an average altitude of 1955 m.a.s.l., and the start was at 1991 m.a.s.l., the highest point being at 2020 m.a.s.l. (Figure 1a,b). During the second session, in April, the snow and temperature conditions were changed and, to ensure that experimental conditions were similar to Track A, another route was designed (i.e., Track B). Track B started at 2177 m.a.s.l., with a mean altitude of 2186 m.a.s.l., and the highest point was at 2250 m.a.s.l. (Figure 1c,d). The SAGF handlers suggested and traced the *Endurance* routes taking into account the difficulties that SAGF units commonly encounter in reaching the places of avalanche disasters and the characteristics of the experimental area. Indeed, the *Endurance* exercise was completely comparable to the routine training of the SAGF units (see later for more details). Therefore, this study was carried out without any manipulation or intervention on the human participants (handlers). However, we asked their informed consent to anonymously process distance, speed, and heart rate data collected by their HR/GPS monitor system. 

### 2.2. Subjects 

A sample of 13 units (1 dog and 1 handler per SAGF unit) of the Avalanche SAR Military GdF group were enrolled in the study. The SAGF handlers were all men, aged from 42 to 63 years (Table S1). All of the handlers were physically healthy, as certified by the periodic checks they undergo with a GdF physician. It is important to note that the SAGF handlers work in a military army, where health and fitness are continuously monitored for working purposes, because these handlers are part of rescue forces that must be optimally trained and in optimal health and fit to save other people in very complex and difficult operations, and cannot work if not certified to be in perfect health and fitness to work by the qualified GDF physician. Dogs (SAGF dogs) were ten males and three females, aged from 3 to 9 years, and belonging to various breeds (four Belgian Shepherd Malinois, five German Shepherd, three Border Collies, one mixed breed; Table S2). Their body weight ranged from 24 to 42 kg (mean ± standard deviation = 32 ± 5 kg; median = 33 kg).

The SAGF dogs came from different areas of Northern Italy, where they lived at home with their handlers. All SAGF units were certified to carry out search and rescue missions for at least one year. All SAGF dogs were trained for at least 2 years to become an operative avalanche, surface, and rubble rescue dog, and had to successfully pass the relative search and rescue tests to obtain the certification. Therefore, they became fully operative at approximately three years of age. The training protocol involves several steps and exercises aimed to build psycho-physical fitness and the ability to work in very different and complex contexts. 

The participating SAGF units arrived at Passo Rolle three days before the beginning of the study to allow them to adapt to the new environment. At the Passo Rolle’s School, the SAGF dogs were individually kenneled in indoor pens (2.9 m × 2.1 m × 2.3 m), in a facility adjacent to that of the handlers. All dogs were fed the same commercial, dry dog feed. The feed is provided by the military force and was not modified from their usual diet. None of the handlers claimed to use feed supplements. All SAGF dogs were physically (i.e., found in good health from a veterinarian and X-ray negative for hip dysplasia) and behaviorally tested (i.e., absence of behavioral pathologies assessed by a veterinary behavior consultant) to certify their suitability for search and rescue work. 

All of the SAGF dogs and handlers were routinely health checked by the health personnel of the GDF Military Force, both when working at their regional working station and when at the Passo Rolle SAGF School. The protocols on how to assess the handlers’ health and fitness for physical activity are covered by military protection and privacy; therefore, we cannot report them here. However, we can report that, when the SAGF handlers are training their dogs or when they are carrying out some fitness exercise, such as running, climbing, endurance, or skiing, etc., they always wear a HR/GPS device for monitoring distance, speed, and heart rate responses. These data are routinely checked by the handlers and by the GDF health personnel. Therefore, we have chosen to carry out all experimental trials of this study according to the GdF handlers’ usual training procedures, having constant available radio contact with the health personnel working at the GdF school, who could be called at any time if necessary. 

### 2.3. Experimental Design

**Figure 2:**
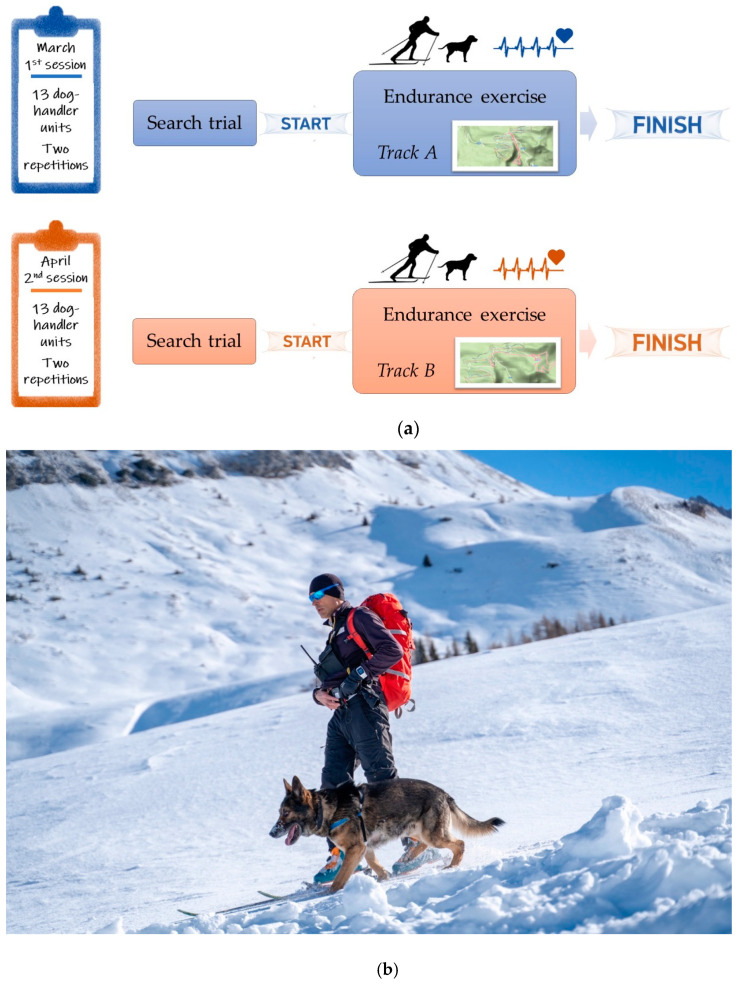
Experimental protocol. Each unit (*n* = 13) performed four Endurance exercises, two following Track A (on two randomly assigned days of the first experimental session, in March), and two following Track B (on two randomly assigned days of the second experimental session, in April). The Endurance exercise started after a search trial and had a maximum allowed time of one hour (panel (**a**)). The SAGF handlers performed the Endurance exercise using ski mountaineering technique while dogs followed them off-leash. During the endurance exercise, SAGF handler and his dog wore the Polar^®^ heart rate monitoring system (panel (**b**)).

The experimental trial consisted of carrying out an *Endurance* exercise soon after a search trial on the designed and tracked Endurance routes (Track A and Track B). Each SAGF unit performed *Endurance* exercise a total of 4 times, twice following Track A and twice following Track B (Figure 2a). Track A was faced during the 1st experimental session, in March, whereas Track B during the 2nd experimental session, in April. The order of the SAGF units in running each trial was randomized by extracting cards with the dog’s name from a box and making sure that each SAGF unit could not repeat the trial twice in the same day. The SAGF handlers performed each *Endurance* exercise using ski mountaineering technique. They were equipped with rescue equipment, including clothes, skis, skins, boots, poles, helmet, backpack, avalanche probe, snow shovel, avalanche transceiver, head lamp, food, drink, etc. (11.4 ± 2.6 kg). The SAGF dogs followed their handlers and were kept off-leash (Figure 2b). The *Endurance* exercise had a one-hour time limit. 

The search trial scheduled before the *Endurance* exercise consisted of discovering a warm scented article buried under the snow, approximately one meter deep, within a field resembling an avalanche fall environment of 25 m × 25 m. More details on the preparation of the field and the odor target have been described by Diverio et al. [15]. The maximum time allowed for the search was 15 min. 

During the *Endurance* activity, both SAGF handlers and dogs were equipped with Polar^®^ System (Polar^®^M400’s built-in GPS, Polar^®^ H7 heart rate sensor, soft elastic belt; Polar Electro Oy, Kempele, Finland). The total weight of the devices was approximately 180 g. Distance, altitude, and HR were registered second by second and transmitted to a computer via USB cable at the end of each recording. All Polar^®^ data were supplied as GPX or CSV files and transferred into Excel spreadsheets.

SAGF handlers positioned their Polar^®^ devices according to the manufacturer’s instructions. The use and reliability of the Polar system for dogs were described earlier [10,21,22]. Before applying the electrodes to the SAGF dogs, their coat was trimmed at all electrode sites, and the skin was cleaned with alcohol and air-dried. Cefar^®^ electrode transmission gel (Cefar-Compex Scandinavia AB) was applied generously to promote conductivity on the cut areas. The electrode belt was strapped ventrally and the electrodes were placed on each side of the sternum [10,22]. A self-adhesive bandaging tape was fixed around the dog’s chest to ensure the device during *Endurance* activity. After Polar^®^ installation, the SAGF dogs appeared to ignore the application of the device, maintaining their natural gait (Figure 2b).

Baseline HR was obtained from each SAGF dog and handler at rest, inside the GdF Alpine School (2001 m.a.s.l.), the day before the trial test. The GdF Alpine School is a familiar environment for dogs and handlers, where they stay frequently to participate in training and refresher courses. Moreover, all subjects were monitored for 15 min, but only a five-minute interval (from min 8 to 13) was extracted and analyzed [10].

### 2.4. Data Processing

Heart rate (HR) provided by the Polar^®^ device second by second was expressed as means ± standard deviation (bpm), highest (HR_max_), and lowest (HR_min_) values. The percentage changes from baseline (Δ% HR) and maximum heart rate (Δ% HR_max_) were also calculated second by second throughout the whole *Endurance* exercise for each dog and were treated as outcome variables. The heart rate deflection point (HRdp) was instead calculated on pooled data and was only used for descriptive purposes. The visual inspection of the relationship between HR and time, as well as the third-order curvilinear (Dmax) methods, was adopted as previously described [10,23] and reported in the supplementary material (Figure S1).

The data obtained from the GPS were treated as factors that could influence HR and, to simplify the presentation and statistical analysis, they were categorized using the criteria summarized in Table 1. Briefly, the altitude information was used to classify the gradient in “level”, “climb”, and “descent” by using the IF function in Excel: if the altitude recorded in a given second was greater than that recorded in the previous 5 s, the gradient was considered “climb”; if lower, “descent”; if equal, “level”. Altitude and speed were categorized in 4 levels according to the information directly provided by the Polar^®^ device (Table 1). To simplify correlation and comparisons analyses, the same ranges were used to categorize the handler’s variables. Finally, the *Endurance* times were monitored by a stopwatch, and the four intervals reported in Table 1 were established.

### 2.5. Statistical Analysis

Statistical analyses were performed using SPSS 25.0 (SPSS Inc., Chicago, IL, USA) and *p* values of <0.05 were considered to be statistically significant.

First, descriptive statistics were used to present the data, calculating the mean and standard deviation (SD), as well as the percentiles of the values (recorded or calculated second by second during the entire *Endurance* activity). Kruskal–Wallis tests were used to compare baseline values of HR between breeds.

Second, the factors that can influence HR during *Endurance* activity were analyzed using multivariable models to evaluate the effects of each factor holding the other variables constant. In particular, Δ% HR and Δ% HR_max_ were included in linear mixed models as dependent variables. “Number of trial” (2 levels: first and second repetition for each track) and “Time” (seconds of the trial) were treated as a repeated measure, whereas SAGF dog or handler was treated as the subject, with a scaled identity covariance structure. The models evaluated several variables as main effects and/or interactions, such as: speed (4 levels: 0 km/h, 0.1–5.4 km/h, 5.5–11.2 km/h, and >11.2 km/h), gradient (3 levels: level, climb, and descent), time intervals of *Endurance* activity (4 levels: 0–15 min, 16–30 min, 31–45 min, and >46 min), track (2 levels: A and B track), number of trial (2 levels: first and second repetition), and breed (3 levels: German Shepherd, Belgian Malinois, and Border Collie). From the breed’s effect analysis, the mixed breed was excluded as only one dog was included. The performance of the search trial could not be included as an independent factor because it was almost constant (i.e., imbalance data; most of the tests, i.e., 79%, were indeed successful). Bonferroni’s correction was used for multiple comparisons. Results were expressed as estimated means ± standard error (SE), while diagnostic graphics were used to check assumptions.

Finally, the relationships between the variables collected in the SAGF dog and the handler were investigated by Pearson’s correlation coefficients (r). Correlation analysis was also performed by stratifying according to the gradient. The correlation was considered poor if r < ∣0.3∣, medium if ∣0.3∣ ≤ r < ∣0.5∣, and large if r ≥ ∣0.5∣ [24].

## 3. Results

### 3.1. Dog’s Heart Rate at Rest, Performance of the Search Trials, and Descriptive Statistics during Endurance Activity 

The mean SAGF dog baseline HR was 97 ± 13 bpm, and it showed no significant differences between breeds (90 ± 9 bpm, 107 ± 14 bpm, and 97 ± 13 bpm in German Shepherd, Malinois, and Border Collie, respectively; *p* = 0.134). 

A total of 52 search trials were carried out before each *Endurance* exercise (26 during the first and 26 during the second session). The warm scented article was found within the time limit in 41 out of 52 search trials (success = 79%).

The average travel times (±standard deviation) of the *Endurance* exercise were 56 ± 5 min. Descriptive statistics for HR, Δ% HR, and Δ% HR_max_ during the *Endurance* exercise are shown in Table 2. The mean HR was 160 ± 34 bpm in the level, 172 ± 34 bpm in climb, and 155 ± 31 bpm in descent. According to speed, HRs were 152 ± 26 bpm, 169 ± 37 bpm, 161 ± 32 bpm, and 156 ± 25 bpm during stationary, the speed of 0.1–5.4 km/h, 5.5–11.2 km/h, and >11.2 km/h, respectively. Finally, the HRdp estimated by the Dmax method resulted after 29–30 min from the start of the *Endurance* activity and corresponded to 70% of HR_max_ (Figure S1). 

### 3.2. Factors Affecting Heart Rate of Dogs during Endurance Activity

The SAGF dogs’ Δ% HR was affected by the gradient (*p* < 0.0001) and speed (*p* < 0.0001). The highest estimated mean of Δ% HR was recorded in climb (76 ± 1%; *p* < 0.0001) and the lowest in descent (61 ± 1%; *p* < 0.0001). On ground level, the values were intermediate (63 ± 1%). The estimated mean of Δ% HR progressively increased as the speed increased. Still, differences between the highest speed categories were not significant (59 ± 1%, 68 ± 1%, 69 ± 1%, and 70 ± 1% in stationary, 0.1–5.4 km/h, 5.5–11.2 km/h, and >11.2 km/h, respectively; *p* < 0.05 except for 5.5–11.2 km/h vs. >11.2 km/h).

**Figure 3:**
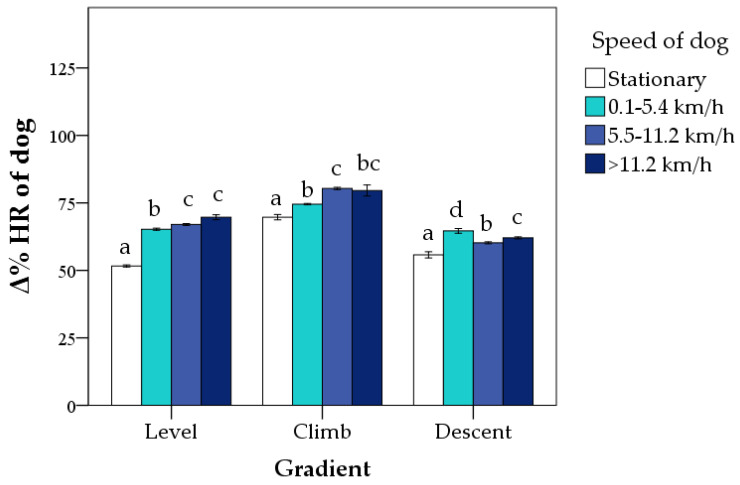
Percentage change from baseline (Δ% HR) of avalanche SAGF dogs (mean ± standard errors) during *Endurance* activity according to gradient and speed. Δ% HR was calculated second by second during the entire period of *Endurance* activity (mean duration = 56 min). Bars not sharing any superscript letter within each gradient are significantly different at *p* < 0.05 (Bonferroni’s correction).

The interaction between gradient and speed was also significant (*p* < 0.0001; Figure 3). On level ground, there was a progressive increase in Δ% HR as the speed increased, although the differences between the highest speeds were not significant. In the climb, the highest speed (>11.2 km/h) only differed from the stationary. In descent, the lowest Δ% HR was recorded during stationary, and the highest during the speed of 0.1–5.4 km/h (*p* < 0.05; Figure 3).

**Figure 4:**
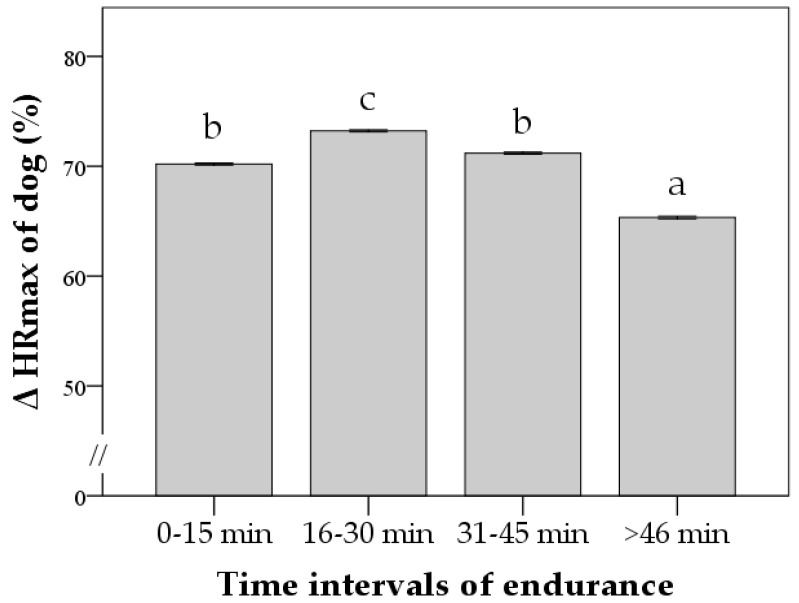
Effect of time intervals of *Endurance* activity on percentage change from maximum heart rate (Δ% HR_max_) of avalanche SAGF dogs. Values are estimated marginal means (±standard errors). The model also included gradient, speed, and gradient x speed. Δ% HR_max_ was calculated second by second during the entire period of *Endurance* activity (mean duration = 56 min). Bars not sharing any superscript letter within each time interval are significantly different at *p* < 0.05 (Bonferroni’s correction).

When time intervals were included in the model, the highest Δ% HR was recorded between 16 and 30 min (73 ± 1%; *p* < 0.001). Then, it decreased, and the lowest values were found after 46 min of *Endurance* activity (56 ± 1%; *p* < 0.0001). Similar results were found for Δ% HR_max_ (Figure 4). A visual inspection of Figure 4 also indicated that HRdp occurred after 30 min of the *Endurance* activity when deflecting the linear relation between HR increase and time.

**Figure 5:**
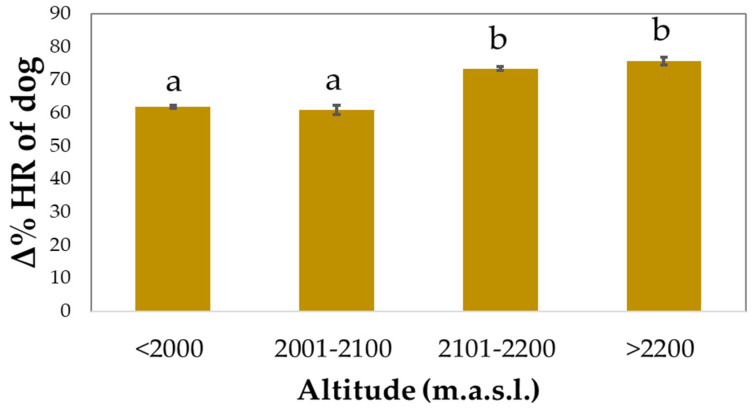
Effect of altitude on percentage change from baseline (Δ% HR) of avalanche SAGF dogs. Values are estimated marginal means (±standard errors). The model also included gradient, speed, and gradient x speed. Δ% HR was calculated second by second during the entire period of *Endurance* activity (mean duration = 56 min). Bars not sharing any superscript letter within each altitude range are significantly different at *p* < 0.05 (Bonferroni’s correction).

There was a progressive increase in the Δ% HR of SAGF dogs as the altitude increased (*p* < 0.0001), but pairwise comparisons showed significant changes only after 2100 m.a.s.l. (Figure 5). Similar results were found for Δ% HR_max_ (not shown). 

With regard to the number of trial and track, the highest Δ% HR was recorded during the second trial (64 ± 0% and 69 ± 0% during the first and second trial, respectively; *p* < 0.0001) and the B track (62 ± 0% and 74 ± 0% during the A and B track, respectively; *p* < 0.0001).

Finally, differences between breeds were also found (*p* < 0.0001): the highest Δ% HR was found for Border Collie (72 ± 1%), followed by German Shepherd (68 ± 1%) and Malinois (61 ± 1%; for all multiple comparisons, *p* < 0.0001).

### 3.3. Handler’s Heart Rate at Rest and during Endurance Activity

The mean HR of SAGF handlers was 79 ± 10 bpm at rest and 156 ± 17 bpm during the *Endurance* activity. The highest value was 193 bpm, whereas the lowest was 77 bpm (Table 2). 

**Figure 6:**
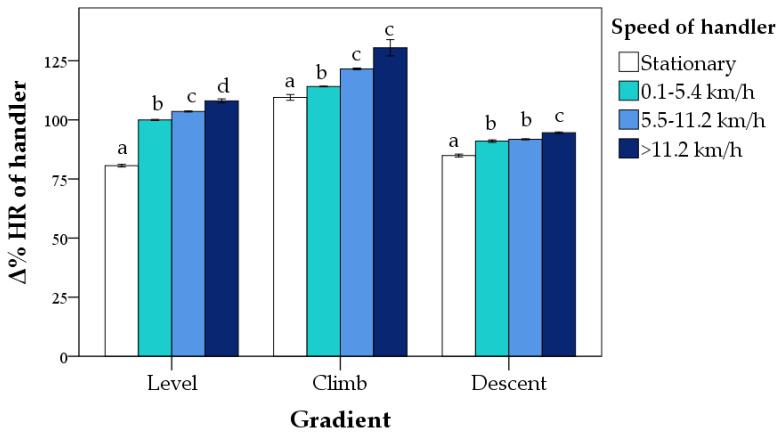
Percentage change from baseline (Δ% HR) of SAGF handlers (mean ± standard errors) during *Endurance* activity according to gradient and speed. The speed of handlers was categorized according to the levels of the dog’s speed. Δ% HR was calculated second by second during the entire period of *Endurance* activity (mean duration = 56 min). Bars not sharing any superscript letter within each gradient are significantly different at *p* < 0.05 (Bonferroni’s correction).

The handler’s Δ% HR was affected by the gradient, speed, and their interaction (for all, *p* < 0.0001). The highest Δ% HR was reached during climbing stages (118.9 ± 1%), the lowest in descent (90.6 ± 0%), and intermediate values in level (98 ± 0%; for all, *p* < 0.0001). With regard to the speed effect, we found progressive increments as they passed from a stationary position (9 2 ± 0%) to >11.2 km/h (111 ± 1%; *p* < 0.0001). Overall, the highest estimated marginal means of the Δ% HR were obtained in the climb at the speed of >11.2 km/h (131 ± 4%; *p* < 0.0001; Figure 6).

With regard to the altitude, the highest Δ% HR was found at <2100 m.a.s.l. (*p* < 0.01; Figure S2). The SAGF handler’s Δ% HR progressively increased throughout the *Endurance* exercise from 91 ± 1% during the first 15 min to 108 ± 1% after 30 min (*p* < 0.0001). Then, the Δ% HR stabilized, and there were no differences after 46 min of exercise (108 ± 1%; Figure S3). We also found significant effects of the number of trial (*p* < 0.0001) and track (*p* < 0.0001): the highest Δ% HR was obtained in the second trial (100 ± 1% and 106 ± 1% during the first and second trials, respectively) and the A track (108 ± 1% and 96 ± 0% during the A and B tracks, respectively).

### 3.4. Association between Heart Rate Changes in the Dog and the Handler

Poor correlations were found in HR changes between SAGF dogs and their handlers. Regardless of the gradient, r was 0.279 and −0.159 for the Δ% HR and Δ% HR_max_, respectively (*p* < 0.01). The lowest coefficients were found when both dogs and handlers moved in descent for the Δ% HR and in the climb for the Δ% HR_max_ (*p* < 0.01; Table 3). 

## 4. Discussion

This study described changes in avalanche dogs’ heart rate collected by a GPS/HR monitoring system during an *Endurance* exercise in an applied environment. The results supported our main study hypothesis showing that the Polar^®^ can capture the HR changes according to the speed of the animal and environmental characteristics, such as the slope and altitude. The device, moreover, was easy to use and reliable, while the dogs did not show any discomfort and did not change their natural gait. These findings suggested that the HR monitor could provide a feasible ABM to optimize avalanche dogs’ training and work–rest cycles, as well as to assess their welfare. Conversely, the second hypothesis of this study must be rejected, as no associations were found between the HR changes in the SAGF dogs and their handler. These findings indicated that the physical effort required of the two members of the SAGF unit during a rescue operation may not be synchronized, even considering the different means they have in covering a trail (the dog on foot and the handler by ski).

The mean HR of SAGF dogs at rest (97 bpm, collected at 2001 m.a.s.l.) was consistent with previous findings on the same breeds (most of the dogs were German Shepherd and Belgian Malinois) [25,26,27]. An agreement with other studies was also found for the HR_max_. Avalanche SAGF dogs reached 239 bpm during the *Endurance* activity. Similar values were recorded by the Polar^®^ system in Australian cattle dogs during mustering exercise (HR_max_ = 237 bpm; [13]) and in Border Collie during treadmill tests (HR_max_ = 230 bpm; [28]). The baseline HR was not affected by the breed. Other studies report conflicting results [29,30], although dogs’ HR differences could be more related to their size than breed. Our animal sample only included medium and large size breeds, with a narrow range of body weights; hence, our results are not conclusive in this regard because there was not enough data variability for reliable comparisons. More interestingly, avalanche SAGF dogs sustained a HR increase of over 70% compared to baseline values for half of the *Endurance* sessions (i.e., one hour; median Δ% HR = 72%). A similar remarkable performance was obtained in other dogs working in challenging contexts, such as cattle or sled dogs [13,31].

To the best of our knowledge, no studies have described the avalanche dogs’ HR during endurance exercise. Moreover, the combined effect of several environmental variables on HR was evaluated for the first time.

First, we described the changes in the Δ% HR of dogs according to the speed and gradient, as well as their interactions. As expected, the Δ% HR progressively increased from stationary (+59%) to the highest speeds (+70%) and from descent (+61%) to level (+63%) and climb (+76%). The linear relationship between HR and speed, at least up to a certain speed range, confirmed the data by Hampson et al. [13] on working cattle dogs. However, the interaction effect showed that the relationship between HR and speed was not always linear and could assume different trends as a function of the gradient. During uphill stretches, there were no differences in HR due to speed, whereas in descent, the highest increase from baseline HR was surprisingly found during the lowest speed. HR is an index of cardiovascular workload and studies on horses support the idea that it could be influenced by several factors altering its linear relationship with speed [32,33]. For example, Voss et al. [34] investigated the horses’ HR during underwater treadmill exercises, but they included the effect of the water at different heights (above the carpus and above the elbow), creating different workloads. They showed that the HR increased from more manageable workloads to heavier ones, which depends on both the gait and water level. The present study confirmed that the workload in a realistic setting is multifactorial, as the HR was affected by non-linear interactions between speed and gradient, as well as duration and other environmental conditions. These findings suggest that a fitness assessment in working dogs should require trials in the field in addition to treadmill tests. A “fitness index” similar to that proposed for horses [12] could be developed, combining HR, speed, and gradient measurements. In particular, it might be useful to understand when a search and rescue dog is “fit to compete”, how it responds to different training methods, and what issues are problematic in the real working environment. Then, SAR handlers could better target training strategies to optimize their dog’s performance.

The SAGF dogs’ Δ% HR significantly increased at the altitude of 2100 m.a.s.l. This result was expected as an adaptation to facilitate O_2_ delivery [35,36]. However, physiological adaptations to altitude could affect the working ability of SAR dogs. Indeed, hypoxia is accompanied by a lowered O_2_ delivery to tissues and the release of reactive oxygen/nitrogen species (RONS) that negatively affect muscle metabolism and contraction [37]. Grandjean et al. [38] evaluated the effect of nutrition on altitude adaptation in SAR dogs, showing that exposure to high altitude enhanced the HR at rest and after a search trial, whereas it decreased the work efficiency. They also showed that supplementation with fish oil and vitamin E improved the adaptability of these dogs, probably through an enhanced oxygen uptake by muscle cells and reduced oxidative stress. Thus, the health of dogs working at high altitudes should be closely monitored and the use of specific supplements to reduce the risk of RONS production may be suggested.

Holding a constant gradient and speed, an increase in the avalanche SAGF dogs’ Δ% HR from the start to 30 min of the *Endurance* activity was found, indicating a progressive decrease in the cardiac vagal tone. After 30 min, conversely, the Δ% HR was reduced. This pattern could suggest that, after 30 min, the dogs reached the so-called “heart rate deflection point” (HRdp). The HRdp determined through a mathematical approach confirmed this estimate. The HRdp is a downward or upward change from the linear HR–work relationship evidenced during incremental progressive exercise tests [10,23,28]. The HRdp has been used as a measure of the capacity to perform at a high intensity for a prolonged period, and it is related to the endurance performance [10,23,28]. Although the physiological mechanisms involved in the occurrence of the HRdp are yet to be fully elucidated, Conconi et al. [39] proposed that it is caused by the activation of anaerobic metabolism. The HRdp may depend on several factors, such as the breed, body weight, protocol, age, excitement, and training [10,23,28,33,40]. The *Endurance* tracks evaluated in the present study did not necessarily involve a progressively increasing workload as required by Conconi’s test [23]. However, we could assume that the workload capable of determining the HRdp in our avalanche dogs began after 30 min of the *Endurance* activity, when the HR was 70% of its maximum values. Further studies would allow HRdp to be applied to the selection and training purposes of SAR dogs. For example, it could be used as a noninvasive marker of exercise intensities to build individualized training programs based on increasing workloads, and to indicate changes in dogs’ physical fitness or welfare status [10,23]. 

The track also influenced the HR. The findings indicated that the B track was more challenging than the A track for the SAGF dogs because it led to a greater Δ% HR. Interestingly, the opposite was found for the SAGF handlers. This could suggest a different perception of the track’s difficulty between dog and handler. This might be due to the different means they use to travel on the *Endurance* track routes (the dogs on foot and the handlers on ski). However, the clinical significance of this discrepancy was marginal, as differences in Δ% HR were lower than 10%. Similarly, we consider no clinically significant differences between the first and the second *Endurance* exercise (i.e., repetition; approximately 5%). 

As mentioned above, the hypothesis of the association between the SAGF dog’s and handler’s HR during an *Endurance* activity in the applied environment was refuted. The correlation between the two values was, indeed, poor, and it became even weaker when they faced an uphill or downhill stretch. These differences are likely due to the fact that the handler was skiing whereas the dog was running, which results in different amounts of effort. Thus, the handler may be underestimating their dog’s fatigue, especially in the downhill sections. Surprisingly, the handlers did not significantly increase their HR compared to baseline when climbing above 2100 m.a.s.l. Few authors have studied the synchronization between dog and human physiological parameters [16,17,18,20]. These previous works mainly evaluated the human–dog correlations of hormone levels concerning changes in emotional states [17,18]. Recently, Carballo et al. [41] demonstrated that handler stress influenced not only the behavior but also the HR of working dogs during search and rescue tasks. In our study, HR changes were primarily related to physical effort, but an influence of the dog and handler’s emotional state could not be excluded, as the monitored activity started after a search trial. Thus, dogs that had failed the search trial could be frustrated and affected by the handler’s stress, whereas those that had found the olfactory target could be excited and perceive the satisfaction of their handler. How did this affect HR? How long? In the present study, most of the search trials were successful. This led to an imbalance of the data that could compromise the reliability of the analyses. Therefore, further studies are needed to discover possible associations between the rescue dog’s and handler’s HR during and after an avalanche search.

Our research may have some limitations. Implementing a field test, indeed, has both strengths and weaknesses. Field testing in avalanche SAR dogs is more representative of the working context, yet it makes it difficult to standardize the many variables that can influence SAR dogs’ physiological responses. Although our study considers the interactions of several variables in HR changes, others remain unexplored. For example, it would be interesting to study the effect of air temperature and snow conditions. Unfortunately, in the field, these parameters might change suddenly from one stretch of the track to another and from one moment to the next in the day. Thus, air temperature and snow conditions could not be included in statistical models. The effect of the breed, sex, size, and body condition, as well as the age and experience level, instead, could not be examined because our sample was not sufficiently variable for these analyses. Moreover, it would be interesting to evaluate the effects of the performance obtained in the search trial. These investigations were beyond the scope of our study, and a larger sample size or different search trials would be required for the creation of two balanced experimental groups (i.e., success vs. failure in the search trial groups). Finally, although several efforts were made to standardize the collection of baseline HR values, it was not possible to control the bias due to the presence of unfamiliar operators and the Polar^®^ device.

## 5. Conclusions

The present study described the changes in the heart rate of dogs and handlers during an *Endurance* exercise in fresh snow, and their influencing factors, by using an HR/GPS monitoring system. The Polar^®^ device captured expected HR changes based on the gradient, speed, and duration of the trial, but also showed that the intricate relationships between these variables influenced the workload. It also indicated that an altitude of 2100 m might be the critical threshold for an increase in the SAR dog’s HR. These results suggested that the Polar^®^ system could be a valuable tool to monitor the heart rate of avalanche SAR dogs in a realistic work environment. The lack of correlation between the dog’s and handler’s HR was another noteworthy finding, as it suggests that the handler may not perceive their dog’s physical conditions in real-time. Increased handler training on animal physiology, as well as the definition of the individual potential of the SAR dogs, could help to manage their physical stress and safeguard their well-being during avalanche rescue efforts. Understanding which characteristics contribute to the success of working dogs, including environmental features, dog’s physical characteristics, the dog–handler relationship, and behavioral traits, should be further investigated to improve the search performance and animal welfare.

## Figures and Tables

**Table 1:** Criteria used to classify the evaluated parameters in dogs and handlers.

Parameter	Classification	Criteria
Gradient	Climb	Altitude recorded in a given second was greater than that recorded in the previous 5 s
	Descent	Altitude recorded in a given second was lower than that recorded in the previous 5 s
	Level	Altitude recorded in a given second was equal to that recorded in the previous 5 s
Altitude	<2000 m.a.s.l.	Altitude provided second by second by the Polar^®^
	2001–2100 m.a.s.l.	
	2101–2200 m.a.s.l.	
	>2201 m.a.s.l..	
Speed	0 km/h (Stationary)	Speed provided second by second by the Polar^®^
	0.1–5.4 km/h	
	5.5–11.2 km/h	
	>11.2 km/h	
Time intervals of *Endurance* activity	0–15 min	From the start (time 0) up to minute 15
	16–30 min	From minute 16 to 30
	31–45 min	From minute 31 to 45
	>46 min	From minute 46 to the end

**Table 2:** Descriptive statistics of the heart rate (HR), percentage change from baseline (Δ% HR), and maximum heart rate (Δ% HR_max_) of avalanche search and rescue SAGF dogs and handlers during *Endurance* activity (recorded or calculated second by second during the entire period of the *Endurance* exercise; duration of *Endurance* activity = 56 ± 5 min).

Subject	Parameter	Mean ± SD	Range	Percentile					
				05	25	50	75	95	99
Dog	HR (bpm)	163 ± 34	27–239	105	143	164	187	217	231
	Δ% HR (%)	68.2 ± 35.0	−77.2–193.8	2.4	48.5	72.2	89.2	121.1	150.0
	Δ% HR_max_ (%)	70.3 ± 14.1	11.5–100	44.9	62.0	71.4	80.0	91.9	97.5
Handler	HR (bpm)	156 ± 17	77–193	128	146	158	167	182	187
	Δ% HR (%)	103.6 ± 28.6	7.5–166.1	59.8	81.5	102.3	125.0	150.8	161.2
	Δ% HR_max_ (%)	88.9 ± 8.03	48.8–100	73.6	84.7	91.1	94.8	98.3	99.4

SD = standard deviation

**Table 3:** Pearson correlation coefficients between Δ% HR and Δ% HR_max_ values of avalanche SAGF dog and handlers stratified according to gradient (calculated second by second during the entire period of *Endurance* activity).

Parameter	Gradient		
	Climb	Descent	Level
Δ% HR	0.251 **	0.196 **	0.242 **
Δ% HR_max_	−0.038 **	−0.057 **	−0.089 **

** *p* < 0.01; Δ% HR = percentage change from baseline; Δ% HR_max_ = percentage change from maximum heart rate

## Data Availability

The data presented in this study are available in the article and Supplementary Materials. Further information is available upon request from the corresponding author.

## Supplementary Materials

The following supporting information can be downloaded at: https://www.mdpi.com/article/10.3390/ani12020168/s1, Table S1: Demographic characteristics of dogs; Table S2: Dogs′ demographic characteristics; Figure S1: Effect of altitude on %Δ HR of handlers; Figure S2: Effect of time intervals of *Endurance* activity on %Δ HR of handlers; Figure S3: Effect of time intervals of endurance on the percentage change from baseline (%Δ HR) of handlers.
