# Supplementary material for: How Do Avalanche Dogs (and Their Handlers) Cope with Physical Exercise? Heart Rate Changes during Endurance in a Snowy Environment
Source: Animals (Basel). 2022 Jan 11;12(2):168. doi: 10.3390/ani12020168 (PMC8772580; doi:10.3390/ani12020168)
Supplement: Supplementary file 1 [file animals-12-00168-s001.zip › animals-1477746-supplementary.pdf]

## Supplementary materials

**Table S1:** Demographic characteristics of dogs.

| ID | Working Area | Age (years) | Experience (years) | Body Weight | Sex |
|---|---|---|---|---|---|
| 1 | Trentino | 48 | 17 | 63 | ♂ |
| 2 | Piemonte | 54 | 28 | 76 | ♂ |
| 3 | Piemonte | 51 | 27 | 70 | ♂ |
| 4 | Trentino | 49 | 25 | 78 | ♂ |
| 5 | Veneto | 43 | 10 | 61 | ♂ |
| 6 | Val d'Aosta | 54 | 30 | 68 | ♂ |
| 7 | Trentino | 63 | 32 | 72 | ♂ |
| 8 | Trentino | 54 | 29 | 95 | ♂ |
| 9 | Friuli Venezia Giulia | 54 | 17 | 72 | ♂ |
| 10 | Trentino | 52 | 25 | 73 | ♂ |
| 11 | Trentino | 54 | 15 | 78 | ♂ |
| 12 | Trentino | 42 | 10 | 78 | ♂ |
| 13 | Piemonte | 46 | 10 | 65 | ♂ |

**Table S2:** Dogs' demographic characteristics.

| ID | Breed | Age (years) | Body weight (Kg) | Sex | Neutering |
|---|---|---|---|---|---|
| 1 | German Shepherd | 9 | 29 | ♀ | No |
| 2 | Belgian Malinois | 8 | 34 | ♂ | No |
| 3 | Belgian Malinois | 8 | 27 | ♀ | No |
| 4 | Belgian Malinois | 4 | 30 | ♂ | No |
| 5 | German Shepherd | 7 | 34 | ♂ | No |
| 6 | Border collie | 3 | 24 | ♂ | No |
| 7 | Mixed bred | 3 | 42 | ♀ | No |
| 8 | Border collie | 5 | 27 | ♂ | No |
| 9 | German Shepherd | 9 | 35 | ♂ | No |
| 10 | German Shepherd | 5 | 38 | ♂ | No |
| 11 | Belgian Malinois | 5 | 33 | ♂ | No |
| 12 | German Shepherd | 7 | 36 | ♂ | No |
| 13 | Border collie | 6 | 29 | ♂ | No |

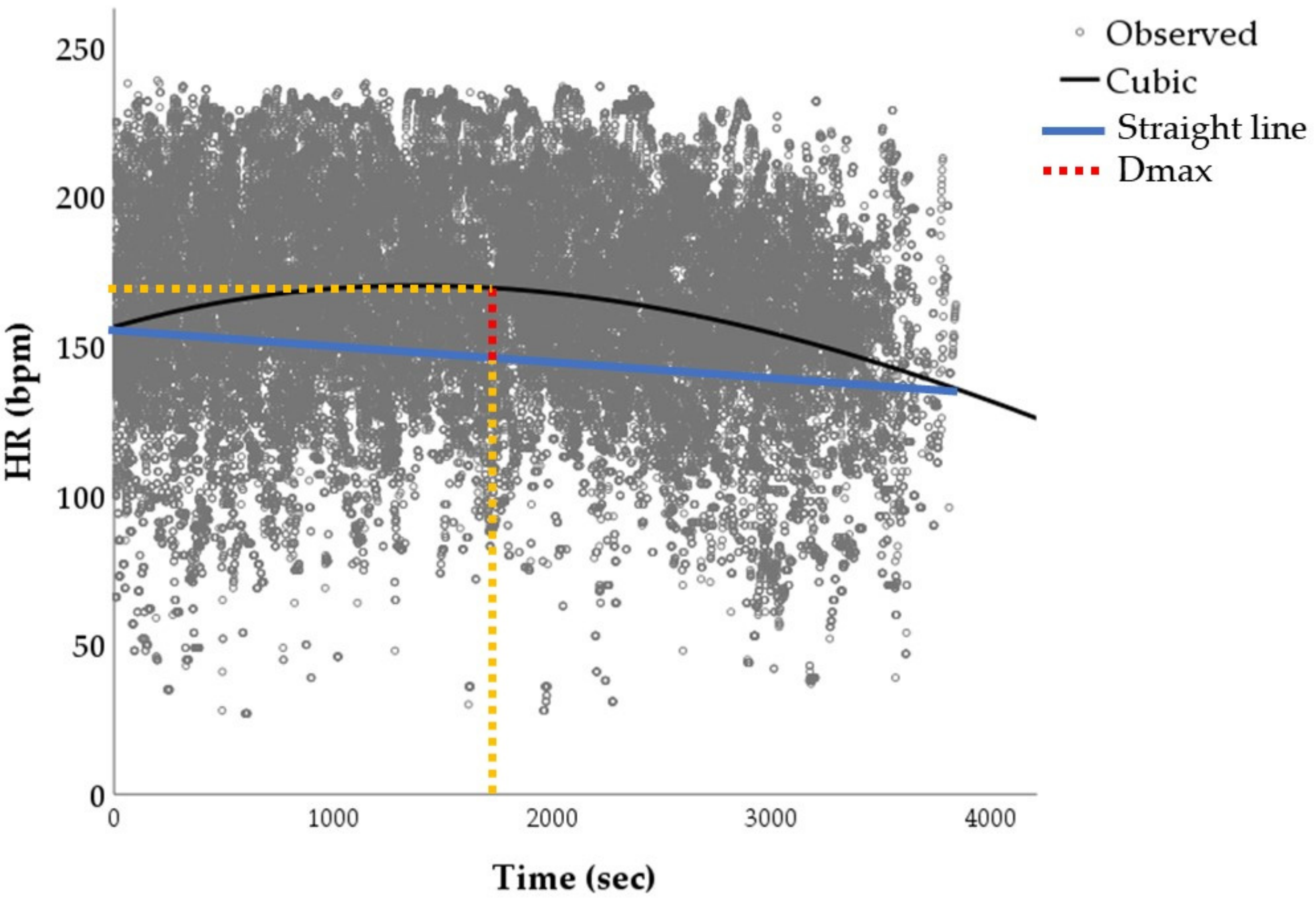


**Figure S1**. Effect of altitude on %Δ HR of handlers. Deflection point (HRdp; dashed line) determined via Dmax method on pooled data. In brief, the third order curvilinear regression curve was calculated from HR values *vs* time (black line). Then, two end points of the curve were connected by a straight line (blue line) and the most distant point of the curve to the line (Dmax; red dashed line) was considered as the HRdp. The HRdp corresponded to the second 1752-1753 of the Endurance activity and an estimated HR of 170 bpm (≈70% of $HR_{max}$).

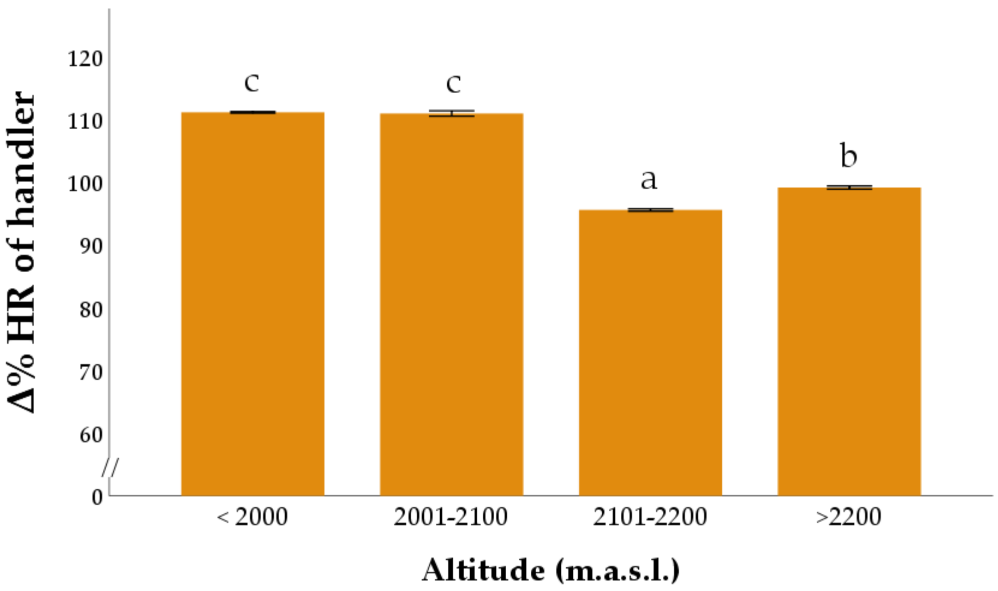


**Figure S2**. Effect of time intervals of *Endurance* activity on %Δ HR of handlers. Effect of Altitude on the percentage change from baseline (%Δ HR) of handlers. Values are marginal means (± standard errors). The model also included Gradient, Speed, and Gradient x Speed. Δ% HR was calculated second by second during the entire period of Endurance activity (mean duration = 56 minutes). Bars not sharing any superscript within each Altitude range are significantly different at $P<0.05$ (Bonferroni's correction).

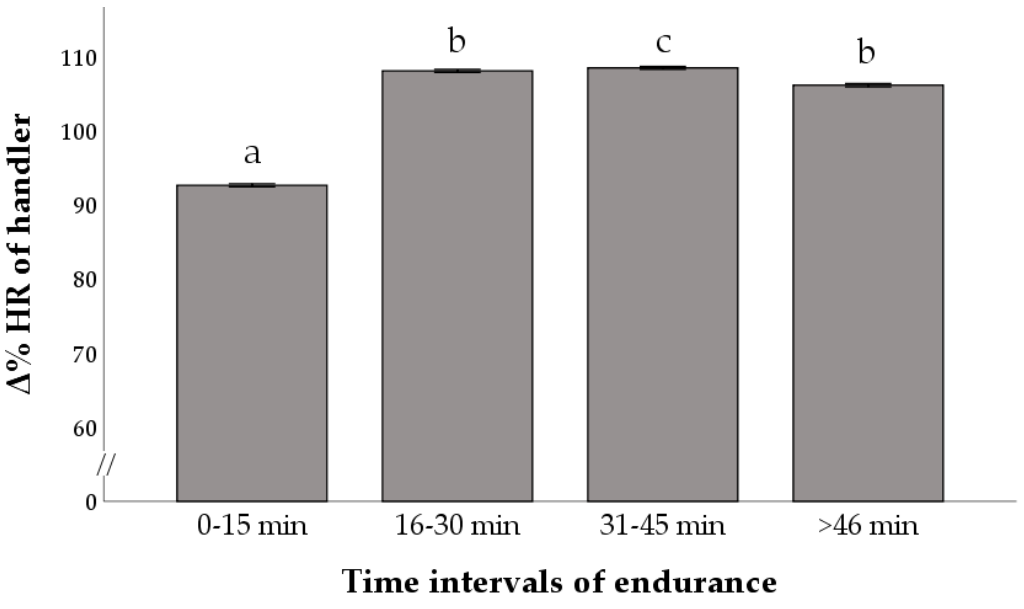


**Figure S3**: Effect of Time intervals of endurance on the percentage change from baseline (%Δ HR) of handlers. Values are marginal means (± standard errors). The model also included Gradient, Speed, and Gradient x Speed. Δ% HR was calculated second by second during the entire period of Endurance activity (mean duration = 56 minutes). Bars not sharing any superscript within each Time interval are significantly different at P<0.05 (Bonferroni's correction).
